# Trends in the Implementation of Active Surveillance for Low-Risk Papillary Thyroid Microcarcinomas at Kuma Hospital: Gradual Increase and Heterogeneity in the Acceptance of This New Management Option

**DOI:** 10.1089/thy.2017.0448

**Published:** 2018-04-01

**Authors:** Yasuhiro Ito, Akira Miyauchi, Takumi Kudo, Hitomi Oda, Masatoshi Yamamoto, Hisanori Sasai, Hiroo Masuoka, Mitsuhiro Fukushima, Takuya Higashiyama, Minoru Kihara, Akihiro Miya

**Affiliations:** ^1^ Department of Surgery, Kuma Hospital, Kobe, Japan.; ^2^ Department of Internal Medicine, Kuma Hospital, Kobe, Japan.; ^3^ Department of Head and Neck Surgery, Kuma Hospital, Kobe, Japan.

**Keywords:** papillary microcarcinoma, thyroid, active surveillance, Kuma Hospital

## Abstract

***Background:*** Active surveillance (AS) of low-risk papillary thyroid microcarcinoma (PMC) was adopted as a management modality in both the Japanese guidelines in 2011 and the American Thyroid Association guidelines in 2015. AS was initiated at Kuma Hospital in 1993 but was not immediately accepted by all physicians. This study investigated the history of acceptance of AS at Kuma Hospital over time. The results should assist in the implementation of AS at other hospitals in Japan and other countries.

***Methods:*** This study included 4023 patients who were cytologically diagnosed with low-risk PMC at Kuma Hospital during the 24-year period between October 1993 and June 2016. The trend in the frequency of AS use over time was analyzed, dividing the 24-year study period into five parts based on the change in frequency of AS use: 1993–1997, 1998–2002, 2003–2006, 2007–2013, and 2014–2016.

***Results:*** The frequency of AS use in the present cohort was 65%. The frequency gradually increased from 30% in 1993–1997 to 88% in 2014–2016, with a slight decrease from 51% in 1998–2002 to 42% in 2003–2006. Until 2007, patients were mostly seen by surgeons, and the frequency of AS use varied remarkably among individual surgeons. Since 2007, the number of patients whose therapeutic strategies are determined by endocrinologists has increased, and the frequency of AS use for low-risk PMC by endocrinologists has been higher than that by surgeons.

***Conclusions:*** At Kuma Hospital, acceptance of AS for low-risk PMC gradually increased over the 24-year study period, but AS was not equally accepted by all physicians. Such variations in the acceptance of AS among individual physicians are also expected to exist in other hospitals. However, due to increasing evidence of the safety and superiority of AS over immediate surgery for this indolent disease, it is expected that AS will gain faster acceptance in other hospitals in Japan and around the world.

## Introduction

The active surveillance (AS) of low-risk papillary thyroid microcarcinoma (PMC) was adopted as a management modality for PMC in the guidelines issued by the Japan Association of Endocrine Surgeons (JAES)/Japanese Society of Thyroid Surgery (JSTS) in 2011 (1) and those of the American Thyroid Association (ATA) in 2015 (2). The AS strategy was first proposed by Dr. Akira Miyauchi at a physicians' meeting of Kuma Hospital as an alternative to the tradition of performing immediate surgery for all PMCs, which in most cases are extremely indolent. At that time, Dr. Miyauchi was an associate professor of surgery at Kagawa Medical University and an affiliate surgeon at Kuma Hospital. The trial started at Kuma Hospital in October 1993 after its approval. Dr. Kanji Kuma, who was president of Kuma Hospital at that time, strongly agreed with Dr. Miyauchi's proposal and actively performed AS himself for low-risk PMC patients.

However, not all of the staff surgeons at Kuma Hospital actively performed AS for PMC patients after the approval of AS. This is not surprising because at that time, there was no direct evidence regarding the safety and appropriateness of AS for low-risk PMC. Along with the publication of many studies from 2003 to the present that demonstrated the promising results of AS—that is, (i) evidence that the incidence of the progression of PMC during AS was low and (ii) evidence that rescue surgery after the observation of progression signs (size enlargement and/or new appearance of lymph node metastasis) was not too late—the number of patients undergoing AS at Kuma Hospital has increased significantly (3–10). However, some surgeons tended to perform immediate surgery for low-risk PMC, even though they approved the AS trial. This study describes the history of AS for low-risk PMC patients treated at Kuma Hospital. It is anticipated that this information will promote quicker acceptance of AS at other hospitals in Japan and around the world.

## Methods

This study included 4023 patients who were cytologically diagnosed at Kuma Hospital as having low-risk PMC during the 24-year period between 1993 and June 2016. The definition of low-risk PMC was papillary thyroid carcinoma (PTC) measuring ≤1 cm with no high-risk features unsuitable for AS, that is: (i) the presence of regional lymph node metastasis or distant metastasis (although the latter is very rare), (ii) fine-needle aspiration biopsy findings suggesting high-grade malignancy, and (iii) tumors that may possibly invade the trachea or recurrent laryngeal nerve. Patients who chose AS were observed with ultrasound examination once a year, unless signs of progression were apparent, such as size enlargement of ≥3 mm compared to the size at AS initiation and/or novel appearance of lymph node metastasis on imaging studies, as previously described (5).

Two options were proposed for the candidate patients with low-risk PMC: AS or immediate surgery. The patients chose their preferred option. Of the 4023 patients with low-risk PMC, 2598 (65%) chose AS and the remaining 1425 (35%) patients underwent surgery within 12 months after the diagnosis. The change in the frequency of AS at Kuma Hospital was retrospectively investigated over the above-mentioned 24-year period. The years between 1993 and 2016 were divided into five periods—1993–1997, 1998–2002, 2003–2006, 2007–2013, and 2014–2016—based on the change in the frequency of AS and on the AS-related events shown in Table 1. In 1998, Akira Miyauchi moved from Kagawa Medical University to Kuma Hospital to accept the vice-president position, and he was appointed president in 2001. The first manuscript demonstrating favorable results of AS was published in 2003 (3). From 2014 onward, several important studies were published on the outcomes of AS at Kuma Hospital (6–12). Table 1 shows the events pertaining to the development of AS for PMC from 1993 to 2017 at the time of this writing.

**Table 1.** Periods and Events Relating to the Active Surveillance for Low-Risk Papillary Thyroid Microcarcinomas at Kuma Hospital, Kobe, Japan

*Year*	*Event*	*Period*
1993	Initiation of AS for low-risk PMC	1993–1997
1997		
1998	Akira Miyauchi appointed vice-president of Kuma Hospital	
2001	Akira Miyauchi appointed president of Kuma Hospital Yasuhiro Ito begins working at Kuma Hospital	1998–2002
2002		
2003	The first report on AS is published (3)	2003–2006
2006		
2007		2007–2013
2010	The second report on AS is published (5) The JSTS/JAES guidelines accept AS as a management approach (2)	
2013		
2014	A third report on the relationship between PMC growth and age is published (6)	2014–2016
2015	Immediate surgery is reported to have a higher incidence of unfavorable events than AS (7)	
2016	An overview of AS by Akira Miyauchi is published (9)	
2017	Results of AS in pregnancy are published (12) A review article by Akira Miyauchi is published (10) An analysis of the difference in costs between AS and surgery is published (8)	

AS, active surveillance; PMC, papillary thyroid microcarcinomas; JSTS/JAES, Japan Association of Endocrine Surgeons/Japanese Society of Thyroid Surgery.

A chi-square test was adopted for comparing variables. A *p*-value <0.05 was considered significant.

## Results

**FIG. 1.**
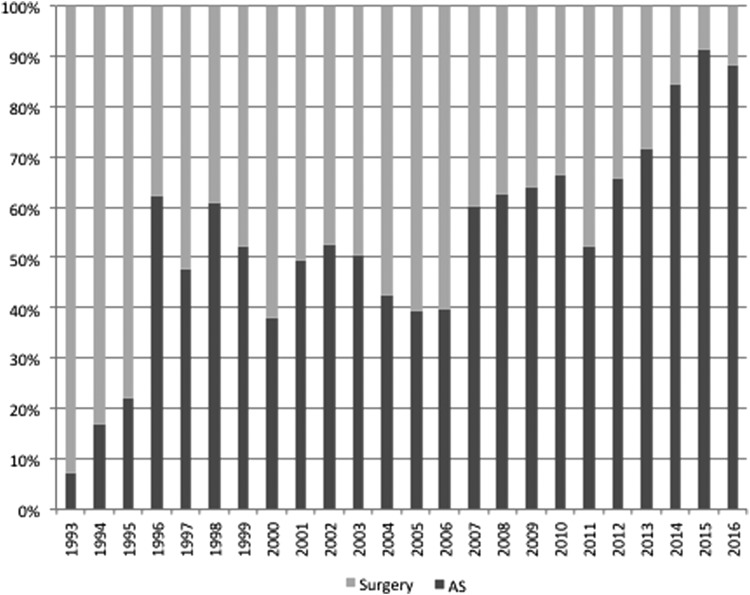
Changes in the frequency of active surveillance (AS) and surgery by year from 1993 to 2016.

**FIG. 2.**
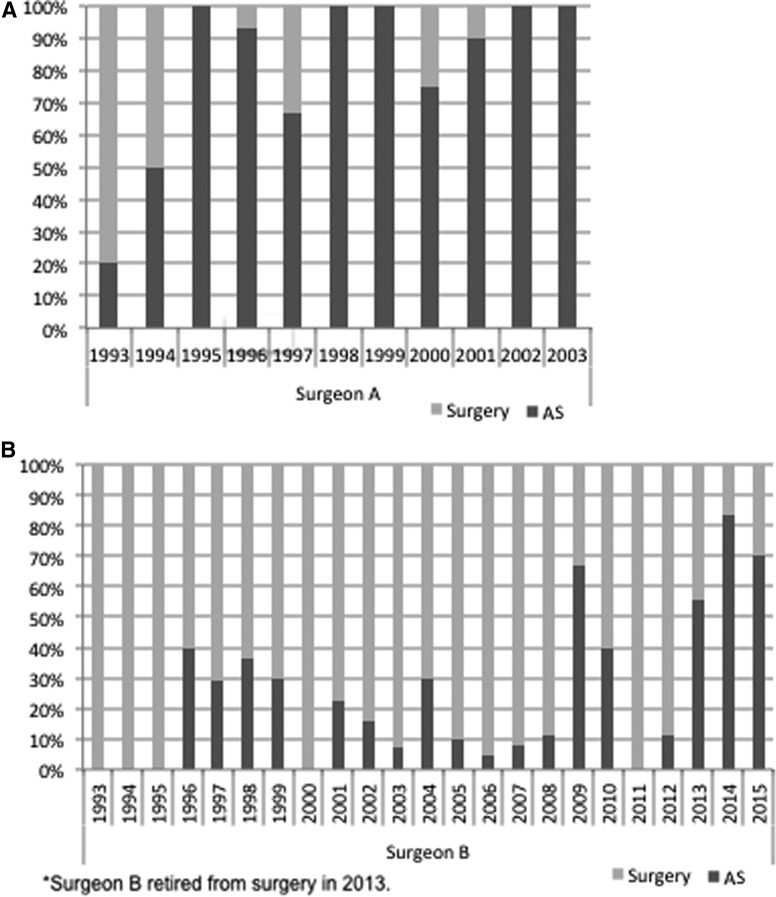

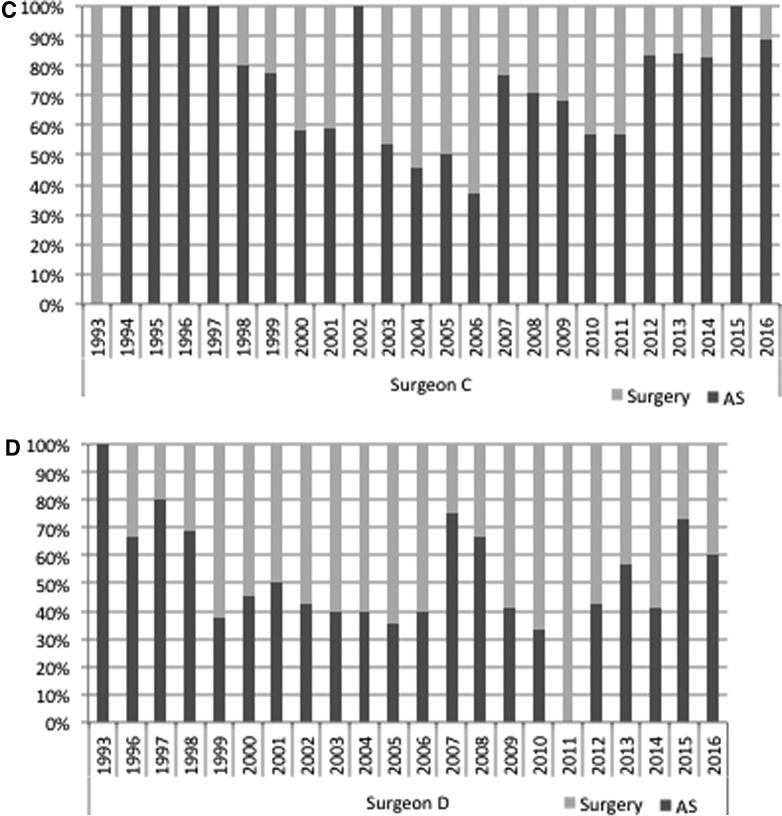

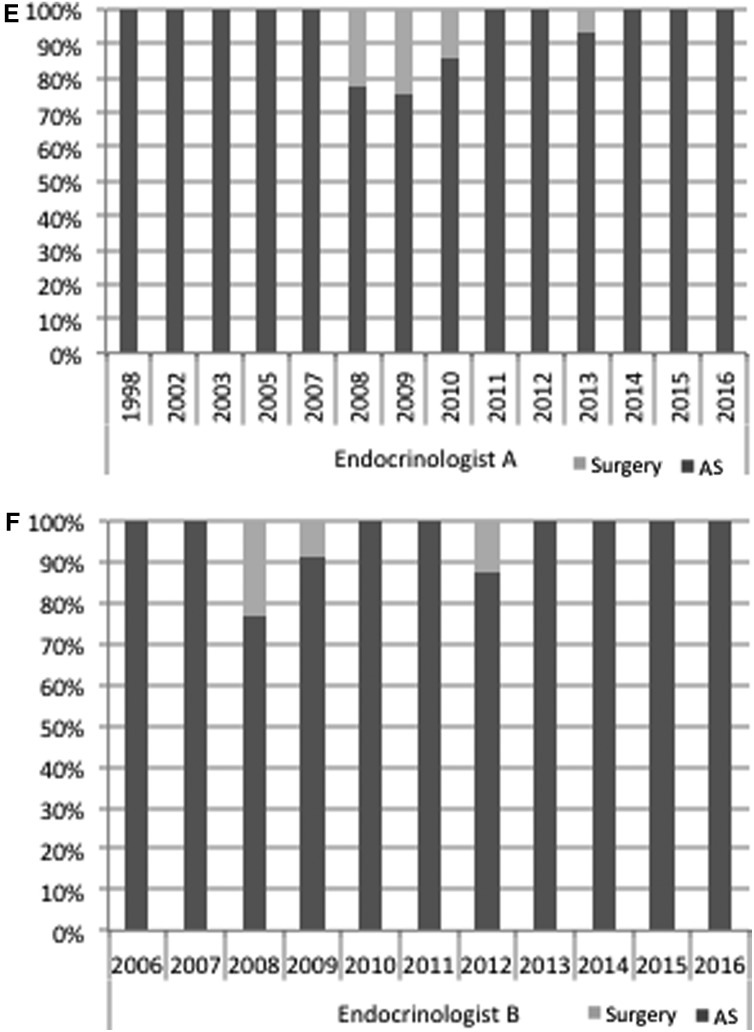
Changes in the frequency of AS and surgery by year and physician. The physicians included in the analysis were four representative surgeons (**A–D**) and two endocrinologists (**E** and **F**) at Kuma Hospital, Kobe, Japan.

Figure 1 shows the trend in the frequency of AS and immediate surgery in each year from 1993 to 2016. The frequency of AS increased from 8% in 1993 to 63% in 1996, but almost plateaued between 1997 and 2002. Then, it gradually decreased from 2003 to 2006, but increased again from 2007 and markedly increased after 2014, reaching about 90%. Figure 2 indicates the change in frequency of AS and immediate surgery in each year for four representative surgeons (surgeons A–D) and two endocrinologists (endocrinologists A and B). There were marked differences in the frequency of AS use among the physicians. Surgeon A performed AS very actively throughout his career (Fig. 2A), whereas surgeon B tended to perform surgery until his retirement from surgery in 2013 (Fig. 2B). The trends in the frequency of AS use by surgeons C and D (Fig. 2C and D) were similar to those for the entire series, as shown in Figure 1, but the frequency of AS use by surgeon C was generally higher than that by surgeon D. Figure 2E and F shows the changes in the frequency of AS use by the two representative endocrinologists. Endocrinologist A and endocrinologist B both used AS frequently throughout the study period.

**FIG. 3.**
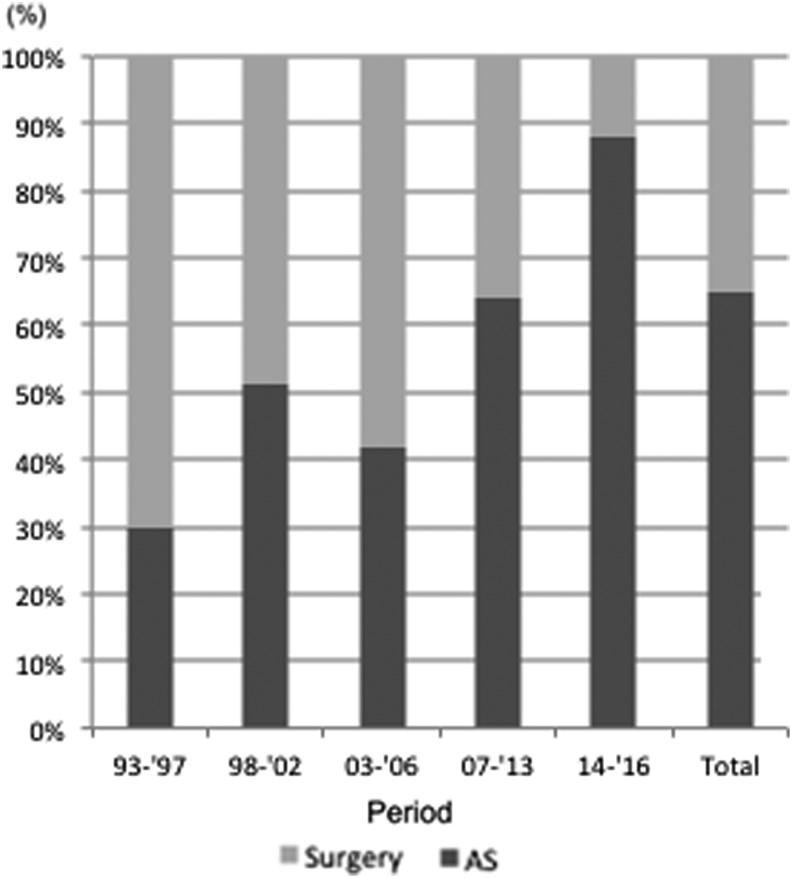
Changes in the frequency of AS and surgery between the intervals 1993–1997 and 2014–2016 at Kuma Hospital, Kobe, Japan.

As the next step in the study, an additional analysis was performed by dividing the study years into five periods, as described in the Methods section, based on the changes in the frequency of AS and on the AS-related events shown in Table 1. Figure 3 and Table 2 indicate the frequency of AS and immediate surgery in each period. The frequency of AS gradually increased from 30% in 1993–1997 to 88% in 2014–2016, except in 2003–2006 when it decreased to 42%. The difference in frequency between intervals was statistically significant (*p* < 0.005) in all cases, except the interval between 1998–2002 and 2003–2006.

**Table 2.** Trends in the Frequency of AS and Immediate Surgery for Low-Risk PMC Over Time at Kuma Hospital, Kobe, Japan

Next, the study looked at differences in the acceptance of AS among surgeons and endocrinologists. The frequency of AS use by the endocrinologists was 86%, which was significantly higher (*p* < 0.0001) than that by the surgeons (58%; Table 3). The trend in the frequency of AS use by surgeons was quite similar to the trend for all cases, as shown in Table 2 and Figure 3. Although the frequency of AS use by endocrinologists was high, the number of patients whose therapeutic strategy was determined by endocrinologists was very small before 2007. The frequency of AS in 2007–2013 by endocrinologists was 79%. The frequency rose and reached 97% in 2014–2016. At Kuma Hospital, most of the patients who were seen by endocrinologists in the earlier periods were referred to surgeons to decide whether to undergo AS or surgery. The number of patients whose therapeutic strategies were determined by endocrinologists increased from 2007 (Table 3), but the endocrinologists still tended to refer cases with risky features to surgeons. This might partially explain the difference in the frequency of AS use between these two groups.

**Table 3.** Trends in the Frequency of AS and Immediate Surgery for Low-Risk PMC by Surgeons and Endocrinologists Over Time at Kuma Hospital

## Discussion

The results of the present analyses demonstrate that (i) the frequency of AS use for low-risk PMC increased along with the accumulation of evidence of the safety and superiority of AS over immediate surgery, (ii) the frequency of AS use by endocrinologists was higher than that by surgeons, and (iii) the frequency of AS use varied among physicians, with some surgeons rarely using AS throughout their careers.

As indicated in the Introduction, at the time of the initiation of the AS trial, there was no direct evidence of the safety and appropriateness of AS, although the following studies strongly suggested that performing surgery for all low-risk PMC could result in more harm than good. In 1994, Takebe *et al*. conducted a thyroid cancer screening study using ultrasound examination and ultrasound-guided fine-needle aspiration cytology on subjects who visited their center for breast cancer screening, and the screening results demonstrated that 3.5% of otherwise healthy Japanese adult women had small thyroid cancer (85% of these were PMC) (13). This incidence was close to those of small latent thyroid cancer in autopsy studies and >1000 times the prevalence of clinical thyroid carcinomas in Japanese women reported at that time (13). This large discrepancy in the incidences strongly suggests that performing surgery for all PMCs might result in more harm than good. Therefore, the decision was made to propose two management options to patients with low-risk PMC: AS or immediate surgery.

In the first period, 1993–1997, the frequency of AS was only 30%, but it increased significantly to 51% in the second period, 1998–2002. This increase may have been related to Dr. Miyauchi's assignment to Kuma Hospital as the vice-president in 1998. After the publication of the first report on AS in 2003, however, the frequency of AS decreased temporarily. It is difficult to identify the reason for this decline, but it may have been partly because no further studies were published following the first one (5). From 2007, the frequency of AS increased again, and after 2014, it rose dramatically to reach 88%. After 2014, several important studies were successively published at Kuma Hospital (6–10). These included a third study on the outcomes of patients who underwent AS (6), which showed an inverse relationship between the progression of PMC and the age of patients. In 2016, Oda *et al*. showed that although the oncological outcomes of AS and surgery as PMC management modalities were similarly excellent, the incidences of unfavorable events were significantly higher in the patients who underwent immediate surgery compared to those who underwent AS. Although all of the surgeries were performed by highly experienced endocrine surgeons at Kuma Hospital, a Center for Excellence in Thyroid Care, significant adverse events such as permanent vocal cord paralysis and permanent hypoparathyroidism could not be completely avoided (7). In 2017, Oda *et al*. also reported that the 10-year total cost of immediate surgery for PMC was 4.1 times the 10-year total cost of AS (8). These three studies provided convincing evidence in favor of AS over immediate surgery, and it is worth noting that all these data were available to the physicians at Kuma Hospital one to two years before their publication. Based on the persuasive evidence described in these recent reports, AS is now recommended as first-line management for patients with low-risk PMC, although the immediate surgery option is still offered for those patients who want it. This policy could account for the striking increase in the use of AS for low-risk PMC patients in 2014–2016.

Another important issue is the distinct difference in the acceptance of AS as a new management modality among physicians. Some physicians, including Dr. Kanji Kuma, accepted AS and frequently recommended it from the beginning. This was very important because if Dr. Kuma had rejected its adoption, AS would not have been performed at Kuma Hospital.

In Japan, unlike in many Western countries, thyroid carcinoma (including diagnosis, treatment, and postoperative follow-up) is predominantly managed by endocrine surgeons rather than endocrinologists. However, at Kuma Hospital, not only surgeons but also endocrinologists initially examine patients with thyroid carcinomas. Some patients are referred to surgeons after the diagnosis of PMC, but many patients are diagnosed with PMC after visiting Kuma Hospital for evaluation of their thyroid nodules. In the early phase, PMC patients chose AS or immediate surgery based mostly on the information given by surgeons, but the proportion of PMC patients whose management decision was based primarily based on an evaluation by and discussion with an endocrinologist increased with time (Table 3). It is interesting that the frequency of AS use by endocrinologists was much higher than that by the surgeons. This may be partially because endocrinologists often refer their PMC patients with concerning features to surgeons, and surgeons often recommended surgery for these cases. It is also possible that there was a decision bias in regard to therapy in low-risk PMC patients; for example, well-experienced surgeons such as surgeon B might have considered surgery for PMCs an easier option and thus tended to recommend immediate surgery to their patients. However, in 2014–2016, the frequency of AS use was similar between patients who were initially examined by an endocrinologist and those initially examined by a surgeon, suggesting that, at the present time, surgeons and endocrinologists are choosing treatment strategies in a similar way.

The history of the application of AS for PMCs following its introduction at Kuma Hospital seems reasonably straightforward. As might be expected, the physicians employed AS at different frequencies in the early period, when evidence for the safety and utility of AS was lacking (Fig. 2). Even after the first AS study was published, the lack of continued reports could have discouraged physicians from adopting this new therapeutic strategy.

A 2017 report from Australia demonstrated a similar reticence to accept AS for low-risk PMC (14). However, that survey study was performed before the recent reports demonstrating the superiority of AS over immediate surgery. Indeed, in the 24 years since the initiation of the AS program at Kuma Hospital, a significant amount of data has accumulated and more than a dozen studies demonstrating favorable results of AS were published at Kuma Hospital (3–12) and the Cancer Institute Hospital (Tokyo) (15–17). A clinical framework for AS has also been proposed in the United States (18).

Even at Kuma Hospital, where AS was proposed and initiated, the implementation of AS required nearly 20 years, and there were considerable differences in the acceptance of AS among physicians. However, as evidence of the safety and superiority of AS over immediate surgery continues to accumulate, it is expected that the acceptance of this management option will occur more quickly and smoothly in other countries, thereby avoiding unnecessary surgery for this mostly indolent disease.
